# Cyclic nucleotide-gated ion channel 6 mediates thermotolerance in Arabidopsis seedlings by regulating nitric oxide production via cytosolic calcium ions

**DOI:** 10.1186/s12870-019-1974-9

**Published:** 2019-08-20

**Authors:** Xuan Peng, Xiaona Zhang, Bing Li, Liqun Zhao

**Affiliations:** College of Life Sciences, Hebei Normal University, Shijiazhuang, 050024 China

**Keywords:** *Arabidopsis*, Cyclic nucleotide-gated ion channel 6, Heat shock, Heat shock protein, Nitric oxide

## Abstract

**Background:**

We previously reported the involvement of nitric oxide (NO) and cyclic nucleotide-gated ion channel 6 (CNGC6) in the responses of plants to heat shock (HS) exposure. To elucidate their relationship with heat tolerance in *Arabidopsis thaliana*, we examined the effects of HS on several groups of seedlings: wild type, *cngc6*, and *cngc6* complementation and overexpression lines.

**Results:**

After HS exposure, the level of NO was lower in *cngc6* seedlings than in wild-type seedlings but significantly elevated in the transgenic lines depending on *CNGC6* expression level. The treatment of seeds with calcium ions (Ca^2+^) enhanced the NO level in Arabidopsis seedlings under HS conditions, whereas treatment with EGTA (a Ca^2+^ chelator) reduced it, implicating that CNGC6 stimulates the accumulation of NO depending on an increase in cytosolic Ca^2+^ ([Ca^2+^]_cyt_). This idea was proved by phenotypic observations and thermotolerance testing of transgenic plants overexpressing NIA2 and NOA1, respectively, in a *cngc6* background. Western blotting indicated that CNGC6 stimulated the accumulation of HS proteins via NO.

**Conclusion:**

These data indicate that CNGC6 acts upstream of NO in the HS pathway, which improves our insufficient knowledge of the initiation of plant responses to high temerature.

**Electronic supplementary material:**

The online version of this article (10.1186/s12870-019-1974-9) contains supplementary material, which is available to authorized users.

## Introduction

Plant growth is governed by many environmental factors, including heat shock (HS), which always causes serious damage to plants. Sublethal heat exposure initiates defensive reactions in cells and prevents them from subsequent lethal heat exposure. For example, HS proteins (HSPs) play an important role in cellular responses under HS conditions. Some HSPs act as molecular chaperones to counteract protein denaturation and aggregation; however, other HSPs, such as ubiquitin and certain proteases, target nonnative proteins for degradation [1]. Thus, the regulation of HSP synthesis is an particularly important issue in plant thermotolerance.

Nitric oxide (NO) is a key signaling molecule that participates in a number of processes throughout the life cycle of plants, including seed germination and dormancy, plant growth and development, flowering, and the interactions of plants with biotic or abiotic stresses [2, 3]. Arginine-denpendent NO synthase (NOS) and nitrite-dependent nitrate reductase (NR) are two important enzymes for NO production in plants. The physiological function of NOS has attracted significant interest. L-arginine, NADPH, and O_2_ are its substrates; they are used to produce NO, NADP^+^, and citrulline [4]. Nitric oxide-associated protein 1 (NOA1), is involved in the regulation of NO levels in *Arabidopsis thaliana* (hereafter, Arabidopsis) [5, 6]. Although the mechanism whereby NOA1 affects NO production in plants has not been throughly elucidated, *noa1*, which exhibits an reduced endogenous NO level, is also a valuable material for studies on NO function in plant.

NR, an essential cytosol-located enzyme for nitrogen assimilation, is also an important source of NO for plants [7]. Under normal conditions, it preferentially reduces nitrate to nitrite [8]. In comparison, under extreme growth conditions, NR can transfer electrons from NAD(P)H to NO_2_^−^ to induce the biosynthesis of NO. NR plays a central role in regulating NO production in response to biotic or abiotic stresses. Two highly homologous NRs, nitrate reductase 1 (NIA1) and nitrate reductase 2 (NIA2), have been identified in Arabidopsis. Although the amino acid sequences of NRs are highly conserved, NIA2 shows greater activity than NR1, accounting for about 90% of the total NR activity in plant cells [9].

As an extensively distributed signaling molecule, NO has been found to increase plant tolerance to many environmental factors, including drought, salinity, heavy metals, and disease [2]. Additionally, we even reported that an increase of NO following extreme heat exposure is crucial for plant acclimation to heat stress [10], inconsistent with a previous findings [11], which might be due to light, age, and temperature effects on NO production, as shown in our previous work [12] (see Supplementary Fig. S3). In a recent review [13], a model was presented that over-accumulation of NO induces heat-sensitive phenotypes (and that can be rescued with NO scavengers), in consistent with our former conclusion [10] that only a moderate NO increase could cause increased heat tolerance (see Fig. 2).

We previously reported that hydrogen peroxide (H_2_O_2_) stimulated the production of NO, which in turn regulated the downstream protein calmodulin3 (CaM3) to promote thermotolerance depending on increased HSP accumulation [12]. Interestingly, our subsequent work showed that H_2_O_2_-induced NO stimulated the activities of antioxidant enzymes so as to eliminate excessive H_2_O_2_, indicating a feedback inhibition between H_2_O_2_ and NO in thermotolerance [14]. Though NO plays an important role in thermotolerance, the precise mechanism underlying its induction remains unclear. Further study of this aspect of HS signaling will enrich our understanding of heat tolerance.

Cyclic nucleotide-gated cation channels (CNGCs) are non-selective cation-conducting channels that are activated by cyclic nucleotide monophosphate or hyperpolarization. In plants, CNGCs function in signaling pathways that may be tied to their ability to conduct calcium ions (Ca^2+^) rather than other cations into plant cells [15]. Ca^2+^ mobilization is a central issue in various plant signaling pathways. The Arabidopsis genome contains 20 expressed CNGC genes that have both distinct and shared biological activities [16]. For example, CNGC14-dependent Ca^2+^ signaling plays a direct role in mediating the early posttranscriptional phase of auxin growth responses in Arabidopsis roots [17]. In comparison, Arabidopsis CNGC2 is associated with a jasmonic acid-induced apoplastic Ca^2+^ influx in epidermal cells [18]. Among the eight Ca^2+^ channels in Arabidopsis (including six CNGCs and two glutamate receptor-like channels), CNGC18 is the only one that is critical for pollen tube guidance [19]. However, the function and regulation of plant CNGCs have not been thoroughly evaluated as major issues in plant science.

CNGCs are also thought to mediate Ca^2+^ signaling in the HS pathway. We found that a heat-activated plasma membrane (PM) Ca^2+^-permeable channel, CNGC6, is involved in the expression of HSP genes and acquisition of thermotolerance in Arabidopsis seedlings [20]. In pollen, AtCNGC16 is essential for heat tolerance during pollen development [21]. Conversely, CNGC2 deficiency results in elevated thermotolerance of plants, indicating its negative role in inducing thermotolerance of Arabidopsis plants [22]. These reports might indicate that some CNGCs in Arabidopsis are stimulated by high temperatures and mediate HS signaling at different growth stages.

Over the past several years, an increasing number of studies have considered a close relationship between NO and Ca^2+^ signaling in plants. Ca^2+^ and NO are well established as universal intracellular second messengers [23]. Studies of plants have shown remarkable overlap in their individual pathways; however, it remains controversial which is downstream of the other. Numerous studies point to a specific role for NO in regulating Ca^2+^ signaling. For example, NO released by NO donors induced a transient rise in cytosolic Ca^2+^ ([Ca^2+^]_cyt_) in *Nicotiana plumbaginifolia* cells [24]. In contrast, some studies have assessed the role of Ca^2+^ in initiating NO signaling. For example, in several plant species, Ca^2+^ and CaM act as cofactors to stabilize plant NOS activity and NO accumulation, suggesting that Ca^2+^ or Ca^2+^-CaM directly interact with a NOS-like enzyme in plants [8]. As yet, the relationship between NO and Ca^2+^ is obscure in plants exposed to high temperatures.

In this study, we used the model plant Arabidopsis to explore the functions of NO and the Ca^2+^-permeable channel CNGC6 in high heat conditions. Our results demonstrate the involvement of CNGC6 in NO signaling as an upstream factor in the HS signaling pathway.

## Results

### Effects of HS on NO accumulation in the seedlings of wild type, *cngc6*, a complemented line, and an overexpression line

NO is a plant signaling molecule that plays a crucial role in the response to many environmental stresses, including HS [2]. Numerous studies indicate a specific role for CNGCs in controlling NO accumulation [25]. To investigate the relationship between CNGC6 and NO in thermotolerance, we first examined endogenous NO accumulation at the seedling stage using wild-type plants, a T-DNA insertion mutant (*cngc6*; SALK_042207), a complementation line (COM12; *cngc6 + CNGC6*), and a *CNGC6* overexpression line (OE8; ecotype Columbia [Col] + *CNGC6*) [20]. Intracellular NO formation was examined with the fluorescent probe 4-amino-5-methylamino-2′,7′-difluorofluorescein diacetate (DAF-FM DA), which can permeate the membrane and be transformed by intracellular esterases into 4-amino-5-methylamino-2′,7′-difluorofluorescein (DAF-FM). It does not directly react with the NO free radical, but rather with nitrous anhydride to yield a highly fluorescent triazole compound [26].

**Fig. 1:**
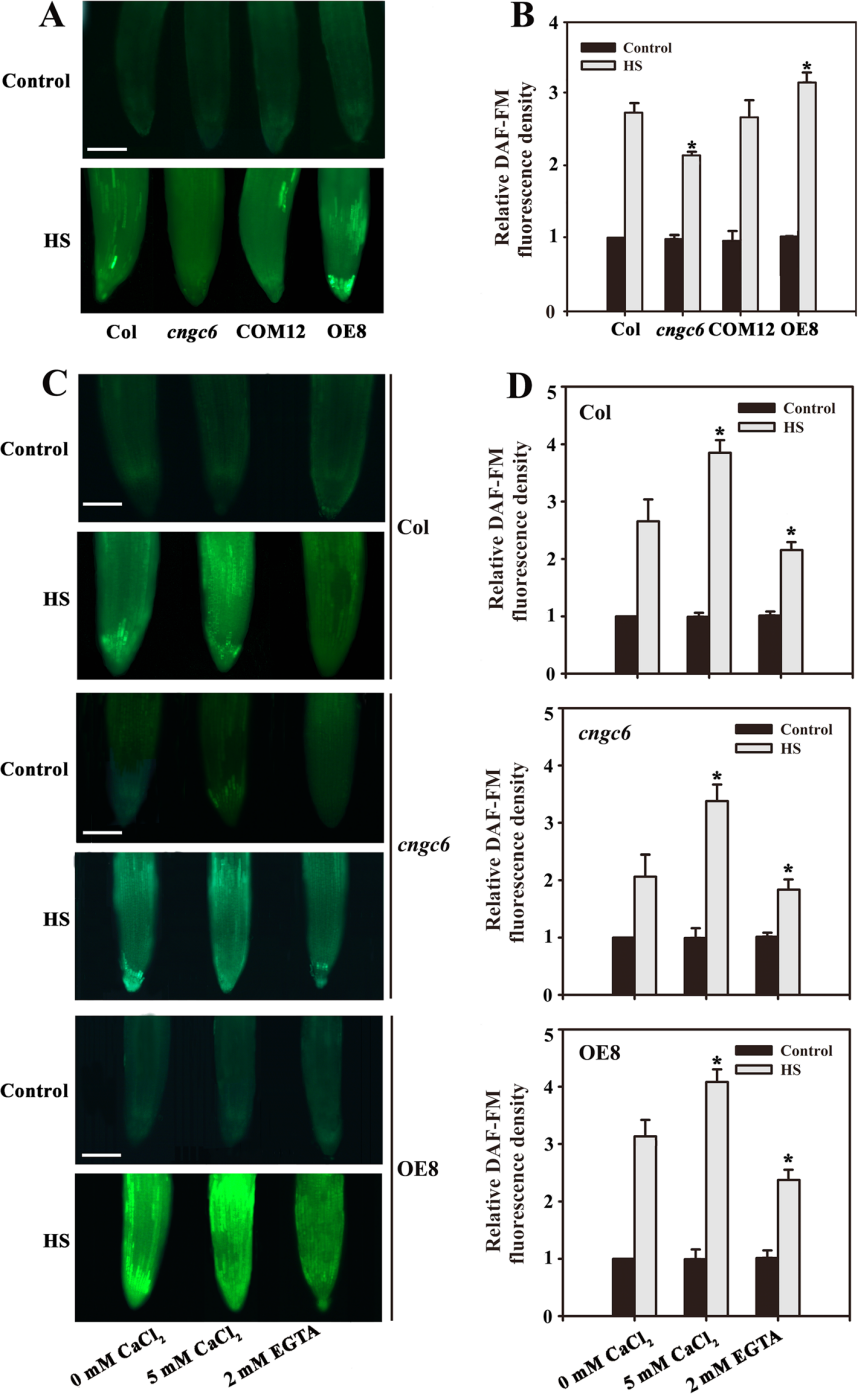
Effects of Ca^2+^ on NO accumulation in Arabidopsis seedlings. (**a**) Eight-day-old wild-type, *cngc6*, COM12, and OE8 seedlings grown at 22 °C were exposed to 45 °C (HS) or maintained at 22 °C (Control) for 60 min. The NO levels in the seedlings were then examined by fluorescence microscopy using roots dyed with DAF-FM DA. Bar = 100 μm. (**b**) Relative DAF-FM fluorescence densities in the roots. The data presented are the means ± standard error (SE) of measurements taken from five independent experiments with at least ten roots for each treatment. *P < 0.05 versus Col (Student’s *t*-test). (**c**) Eight-day-old wild-type, *cngc6*, and OE8 seedlings were exposed to 45 °C (HS) or maintained at 22 °C (Control) for 60 min. The NO levels in the plants were then examined by fluorescence microscopy using roots stained with DAF-FM DA. Bar = 100 μm. (**d**) The relative DAF-FM fluorescence densities in the roots. The data presented are the means ± SE of measurements taken from five independent experiments with at least ten roots for each treatment. *P < 0.05 versus 0 mM CaCl_2_ (Student’s *t*-test)

Fluorescence analysis indicated that under normal growth conditions (22 °C), no clear difference in NO abundance existed among the seedlings. After HS treatment at 45 °C for 60 min [10], the NO level increased by 273% in wild-type seedlings. This is greater than the increase observed in *cngc6* (213%); however, the NO level was nearly completely rescued in COM12 seedlings (269%), and even higher in OE8 seedlings (310%) than in wild-type seedlings (Fig. 1a, b). These data show that the variation in NO observed following HS treatment was dependent on *CNGC6* expression.

### Effect of Ca^2+^ on the NO level in wild-type seedlings

CNGC6 is a heat-activated Ca^2+^-permeable channel in the plasma membrane (PM) of plant cells [20]. Ca^2+^, probably the most versatile ion found in eukaryotes, has been confirmed to coordinate with NO in several physiological processes [8]. Thus, we inferred that CNGC6 promotes the accumulation of NO through Ca^2+^ to mediate thermotolerance.

To verify this hypothesis, we examined the NO levels in wild-type, *cngc6*, and OE8 seedlings that had been pretreated with 5 mM CaCl_2_ or 2 mM EGTA (a Ca^2+^ chelator) before germination [27]. Fluorescence analysis showed no obvious difference in NO levels among the seedlings under normal conditions. Under HS conditions, treatment with 5 mM Ca^2+^ increased the NO level to 389, 326, and 407% of their individual controls, respectively, in wild-type, *cngc6*, and OE8 seedlings. Whereas 2 mM EGTA reduced the increase in NO to 219, 187, and 227% of their individual controls, respectively, in wild-type *cngc6*, and OE8 seedlings (Fig. 1c, d).

### Effects of NO on the thermotolerance of *cngc6* seedlings

Next, we examined the effects of the exogenous application of two NO donors, sodium nitroprusside (SNP) and *S*-nitroso-*N*-acetylpenicillamine (SNAP), on the thermotolerance of *cngc6* seedlings.

**Fig. 2:**
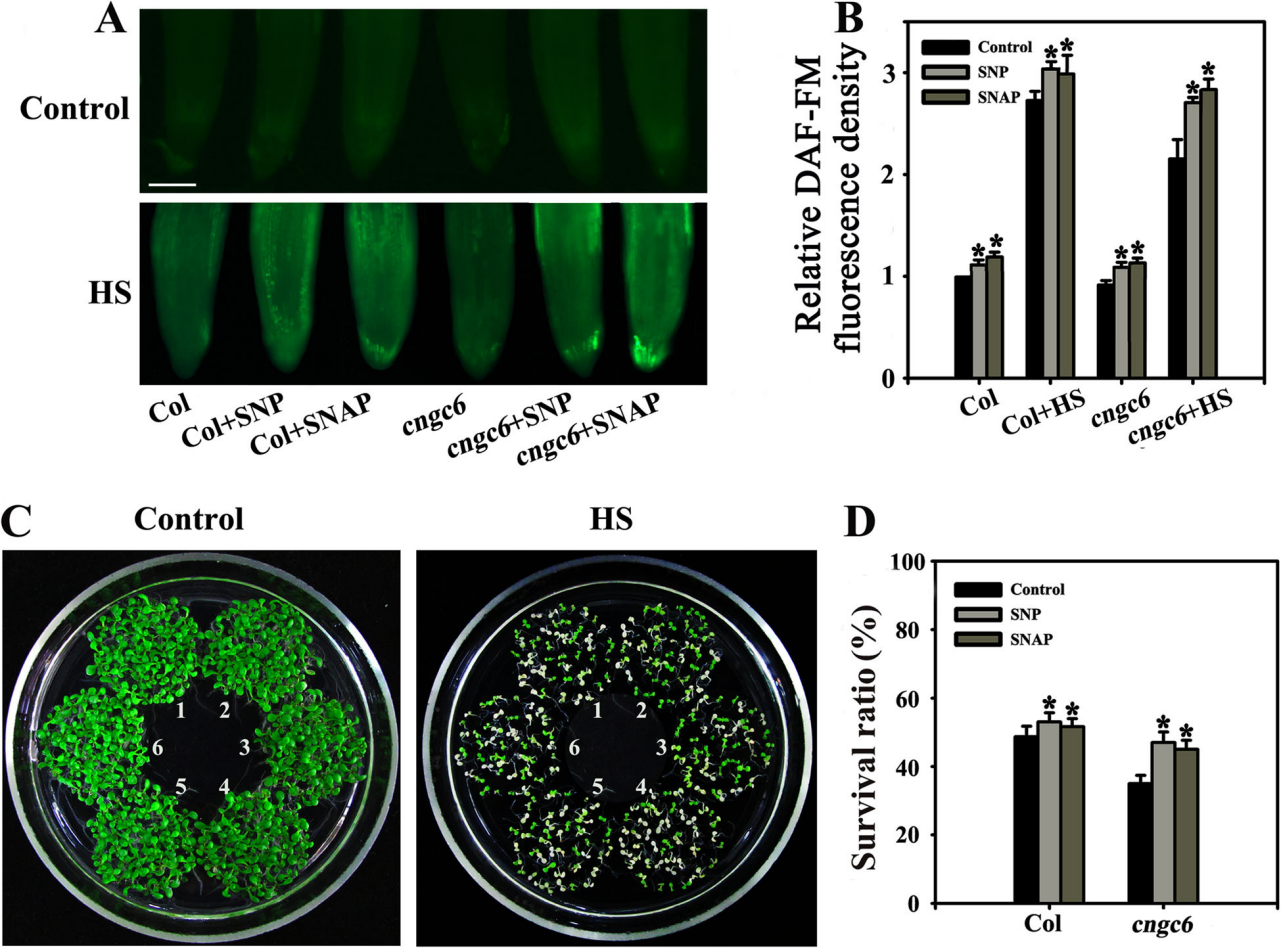
Effects of NO donors on the thermotolerance of *cngc6* seedlings. (**a**) Eight-day-old wild-type and *cngc6* seedlings were exposed to 45 °C (HS) or maintained at 22 °C (Control) for 60 min. The NO levels in the plants were then examined by fluorescence microscopy using roots stained with DAF-FM DA. Bar = 100 μm. (**b**) The relative DAF-FM fluorescence densities in the roots. The data presented are the means ± SE of measurements taken from five independent experiments with at least ten roots for each treatment. *P < 0.05 versus Col. (**c**) Seedlings were exposed to 45 °C (HS) or maintained at 22 °C (Control) for 100 min, then returned to 22 °C and photographed 5 days later. The clusters are as follows: 1, Col; 2, Col + SNP; 3, Col + SNAP; 4, *cngc6*; 5, *cngc6* + SNP; and 6, *cngc6* + SNAP. (**d**) Survival ratios of the seedlings after HS treatment. The data presented are the means ± SE from at least five independent experiments, with 50 seedlings per experiment. *P < 0.05 versus Col (Student’s *t*-test)

**Fig. 3:**
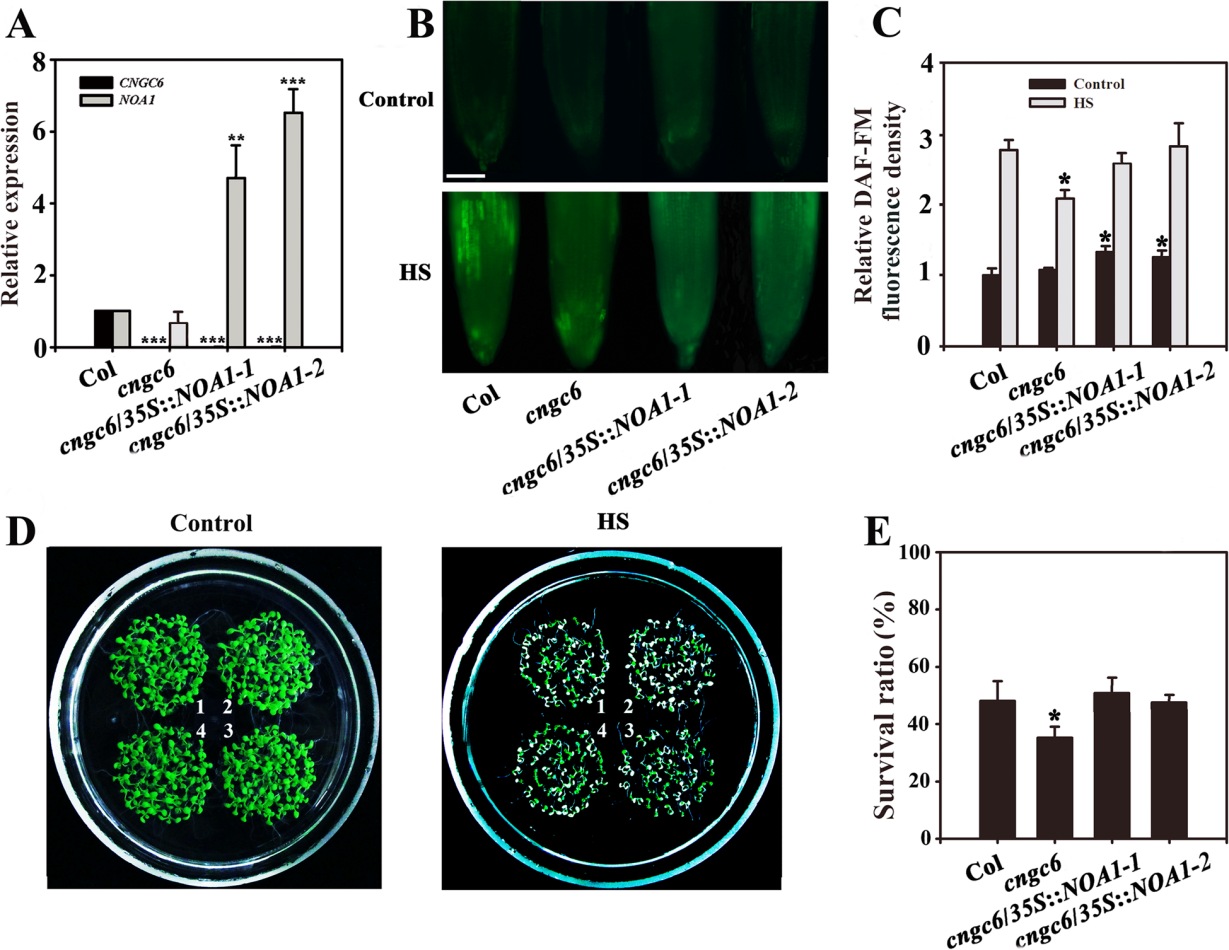
Improved thermotolerance through *AtNOA1* overexpression in a *cngc6* background. (**a**) RT-qPCR analysis of *AtCNGC6* and *AtNOA1* transcription in wild-type, *cngc6*, *cngc6*/*35S*::*NOA1*–*1*, and *cngc6*/*35S*::*NOA1*–*2* plants. The experiments were repeated three times with similar results. Each data point represents the mean ± standard deviation (SD; n = 3). Asterisks indicate a significant difference relative to Col (Student’s *t*-test, **P < 0.01 and ***P < 0.001). (**b**) Eight-day-old wild-type, *cngc6*, *cngc6*/*35S*::*NOA1*–*1*, and *cngc6*/*35S*::*NOA1*–*2* seedlings grown at 22 °C were exposed to 45 °C (HS) or maintained at 22 °C (Control) for 60 min. The NO levels in the plants were then examined by fluorescence microscopy using roots stained with DAF-FM DA. Bar = 100 μm. (**c**) The relative DAF-FM fluorescence densities in the roots. The data presented are the means ± SE of measurements taken from five independent experiments with at least ten roots for each treatment. *P < 0.05 versus Col. (**d**) Seedlings grown at 22 °C were exposed to 45 °C (HS) or maintained at 22 °C (Control) for 100 min, then returned to 22 °C and photographed 5 days later. The clusters are as follows: 1, wild type; 2, *cngc6*; 3, *cngc6*/*35S*::*NOA1*–*1*; and 4, *cngc6*/*35S*::*NOA1*–*2*. (**e**) Survival ratios of the seedlings after HS treatment. The data presented are the means ± SE of at least five independent experiments with 50 seedlings per experiment. *P < 0.05 versus Col (Student’s *t*-test)

Fluorescence analysis revealed that both of SNP and SNAP (20 μM each) increased the amount of NO in wild-type and *cngc6* plants under normal and HS conditions; moreover, the effect was particularly pronounced for SNP (Fig. 2a, b). To measure physiological adaptability to heat stress, survival ratios were calculated for plants following HS treatment at 45 °C for 100 min and 5 days of recovery at 22 °C [28]. Under normal growth conditions (22 °C), no obvious phenotypic difference was observed between wild-type and *cngc6* seedlings (Fig. 2c, Control). After HS treatment, the survival ratio of the wild-type seedlings (48%) was higher than that of *cngc6* seedlings (36%), consistent with our previous report [20] (see Fig. 3). Exogenous pretreatment with 20 μM SNP or SNAP increased the survival ratio of *cngc6* seedlings greatly, to a value that was similar to that seen for wild-type seedlings (Fig. 2d), indicating that the addition of NO rescued the heat sensitivity of the mutant in the absence of CNGC6. Also, it slightly increased the survival ratio of wild-type seedlings (Fig. 2d). Based on these results, we reached the preliminary conclusion that NO is involved in CNGC6 signaling as a downstream factor.

### *AtNOA1* and *AtNIA2* overexpression in a *cngc6* background improves thermotolerance

**Fig. 4:**
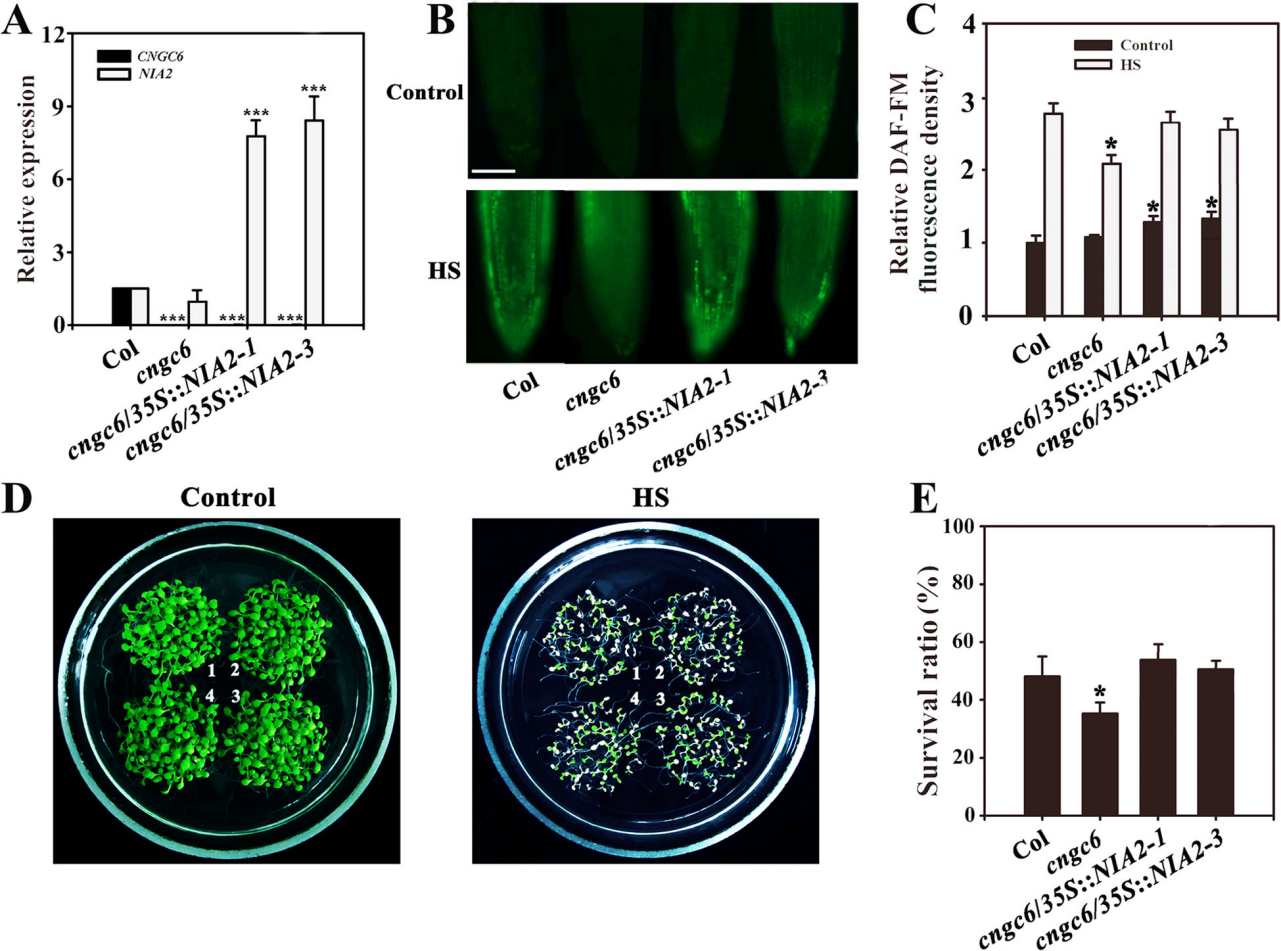
Improved thermotolerance through *AtNIA2* overexpression in a *cngc6* background. (**a**) RT-qPCR analysis of *AtCNGC6* and *AtNIA2* transcription in wild-type, *cngc6*, *cngc6*/*35S*::*NIA2*–*1*, and *cngc6/35S::NIA2–3* plants. The experiments were repeated three times with similar results. Each data point represents the mean ± SD (n = 3). Asterisks indicate a significant difference relative to Col (Student’s *t*-test, ***P < 0.001). (**b**) Eight-day-old wild-type, *cngc6*, *cngc6*/*35S*::*NIA2*–*1*, and *cngc6/35S::NIA2–3* seedlings grown at 22 °C were exposed to 45 °C (HS) or maintained at 22 °C (Control) for 60 min. The NO levels in the plants were then examined by fluorescence microscopy using roots stained with DAF-FM DA. Bar = 100 μm. (**c**) The relative DAF-FM fluorescence densities in the roots. The data presented are the means ± SE of measurements taken from five independent experiments with at least ten roots for each treatment. *P < 0.05 versus Col (Student’s *t*-test). (**d**) Seedlings grown at 22 °C were exposed to 45 °C (HS) or maintained at 22 °C (Control) for 100 min, then returned to 22 °C and photographed 5 days later. The clusters are as follows: 1, wild type; 2, *cngc6*; 3, *cngc6*/*35S*::*NIA2*–*1*; and 4, *cngc6/35S::NIA2–3*. (**e**) Survival ratios of the seedlings after HS treatment. The data presented are the means ± SE of at least five independent experiments with 50 seedlings per experiment. *P < 0.05 versus Col (Student’s *t*-test)

We previously found that NO functions as a signal in thermotolerance using the mutants *noa1* and *nia1 nia2*, which show heat sensitivity due to a deficiency in NO [10]. To further confirm the influence of CNGC6 on NO signaling under HS conditions, we obtained two *AtNOA1*-overexpressing transgenic lines, *cngc6/35S::NOA1–1* and *cngc6/35S::NOA1–2*, and two *AtNIA2-*overexpressing transgenic lines, *cngc6/35S::NIA2–1* and *cngc6/35S::NIA2–3*, and examined the effects of excess internal NO on *CNGC6*-deficient mutants under HS. The elevated expression of *AtNOA1* and *AtNIA2* was confirmed by real-time quantitative RT-PCR **(**RT-qPCR) (Figs. 3a, 4a).

DAF-FM fluorescence analysis revealed that *AtNOA1* and *AtNIA2* overexpression increased the internal NO levels in the transgenic lines under normal and HS conditions (Figs. 3, 4). At room temperature, no obvious phenotypic difference was observed between *cngc6* and the transgenic plants*.* However, at high temperatures, *AtNOA1* or *AtNIA2* overexpression obviously increased the survival ratio of the corresponding transgenic lines compared to *cngc6* (Figs. 3, 4).

These results show that the overexpression of *AtNOA1* or *AtNIA2* restored thermotolerance in a *CNGC6*-deficient mutant, providing genetic proof of the relationship between NO and CNGC6 in thermotolerance.

### Effects of HS on the thermotolerance of *cngc6 noa1* double-mutant seedlings

**Fig. 5:**
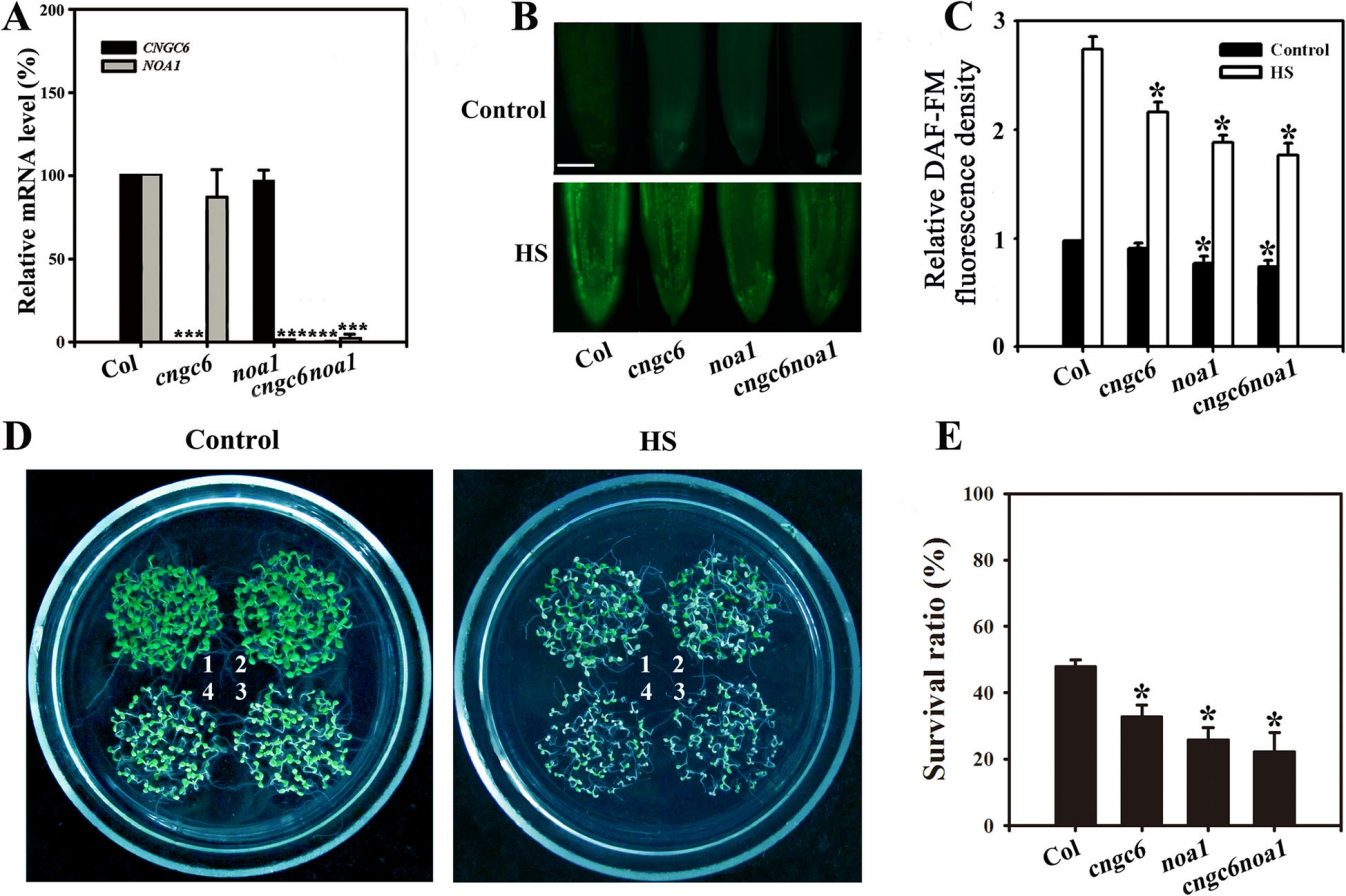
Survival status of the *cngc6 noa1* double mutant. (**a**) RT-qPCR analysis of *CNGC6* and *NOA1* transcription in wild-type, *cngc6*, *noa1*, and *cngc6 noa1* seedlings. The experiments were repeated three times with similar results. Each data point represents the mean ± SD (n = 3). Asterisks indicate a significant difference relative to Col. ***P < 0.001 (Student’s *t*-test). (**b**) Eight-day-old wild-type, *cngc6*, *noa1*, and *cngc6 noa1* seedlings grown at 22 °C were exposed to 45 °C (HS) or maintained at 22 °C (Control) for 60 min. The NO levels in the seedlings were then examined by fluorescence microscopy using roots stained with DAF-FM DA. Bar = 100 μm. (**c**) Relative DAF-FM fluorescence densities in the roots. The data presented are the means ± standard error (SE) of measurements taken from five independent experiments with at least ten roots for each treatment. *P < 0.05 versus Col (Student’s *t*-test). (**d**) Eight-day-old seedlings grown at 22 °C were exposed to 45 °C (HS) or maintained at 22 °C (Control) for 100 min, then returned to 22 °C and photographed 5 days later. The clusters are as follows: 1, wild type; 2, *cngc6*; 3, *noa1*; and 4, *cngc6 noa1*. (**e**) Survival ratios of the seedlings after HS treatment. The data presented are the means ± SE of at least five independent experiments with 50 seedlings per experiment. *P < 0.05 (Student’s *t*-test)

To further determine the roles of CNGC6 and NO in thermotolerance, we obtained the *cngc6 noa1* double mutant by crossing, which was deficient in *CNGC6* and *NOA1* transcription using RT-qPCR (Fig. 5a). Under normal and HS conditions, the NO level in the *cngc6 noa1* seedlings was near to the value in the *noa1* seedlings (Fig. 5b, c), indicating that the absence of *CNGC6* did not obviously decrease NO accumulation in *noa1* seedlings. Under normal conditions, *cngc6 noa1* seedlings showed small and chlorotic, similar to *noa1* seedlings (Fig. 5d, Control). Under HS conditions, the survival ratio of the *cngc6 noa1* seedlings was close to that of *noa1* seedlings (Fig. 5d, e), indicating that the absence of *CNGC6* did not exacerbate the heat sensitivity of *noa1*.

### Effects of CNGC6 via NO on AtHSP17.7 and AtHSP21 expression

**Fig. 6:**
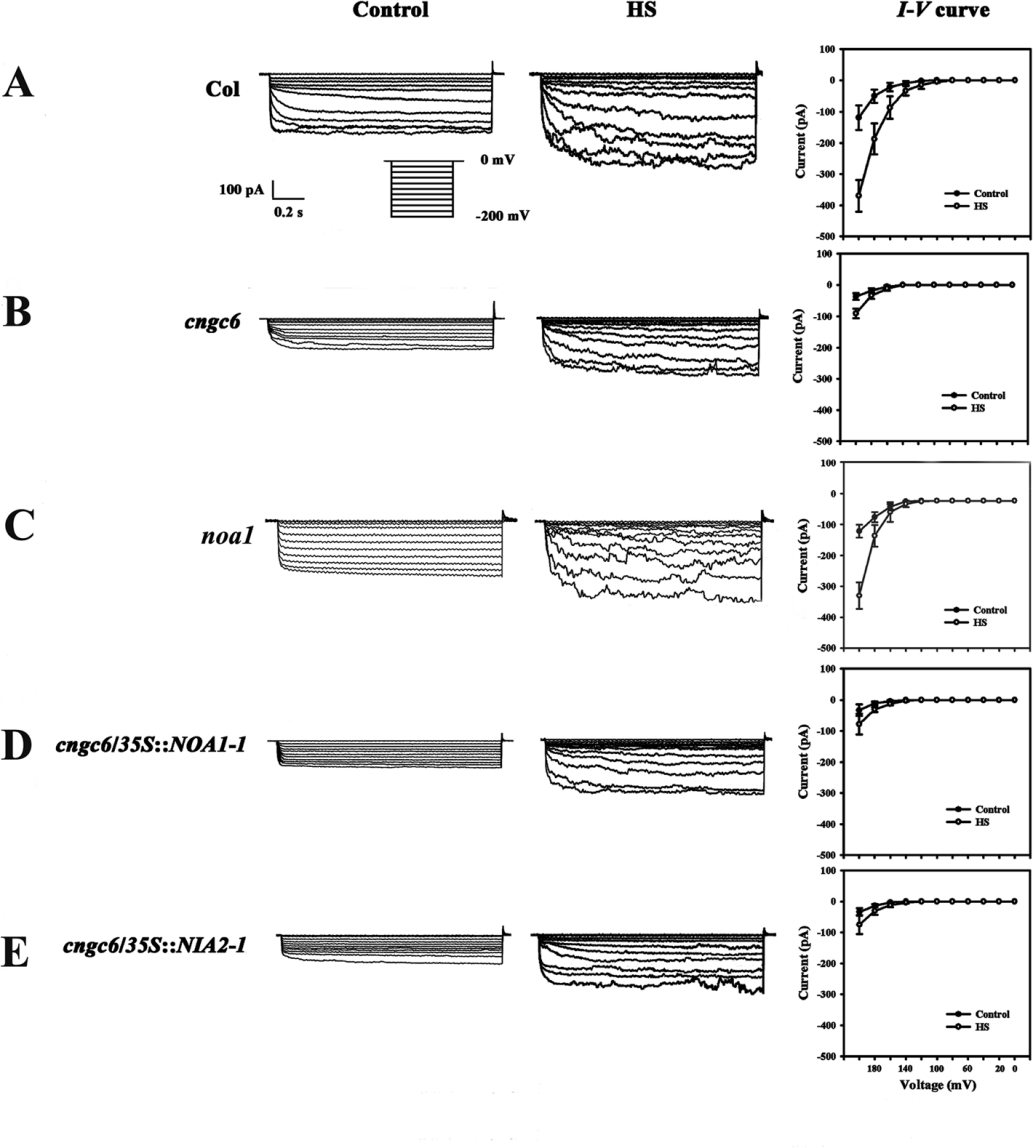
Effects of CNGC6 via NO on AtHSP17.7 and AtHSP21 expression. (**a**–**d**) Ten-day-old seedlings grown at 22 °C were exposed to 37 °C (HS) or maintained at 22 °C (Control) for 2 h. Total protein was then extracted, separated by SDS-PAGE, and analyzed by Western blotting. Tubulin was used as an internal control. Three independent experiments were carried out; the results indicate similar trends in protein accumulation. The numbers below each lane represent the relative intensity of each signal

HSPs are important for all organisms to survive under acute stress by keeping proteostasis as molecular chaperones. To examine the underlying mechanism of CNGC6- and NO-induced thermotolerance in Arabidopsis, we next examined the effects of CNGC6 and NO on the accumulation of AtHSP17.7 and AtHSP21 in seedlings by Western blotting. Both of AtHSP17.7 and AtHSP21 were not detected at 22 °C (Fig. 6a, b); however, the accumulation of both proteins was observed at 37 °C. The level of accumulation was lower in the mutants than in wild type (and lowest for *cngc6 noa1*), and it was strongly stimulated by 20 μM SNP or SNAP. In addition, AtHSP17.7 and AtHSP21 accumulation was stimulated in *cngc6*/*35S*::*NOA1*–*1* and *cngc6*/*35S*::*NIA2*–*1* plants compared with *cngc6* (non-transformed background; Fig. 6c, d). In each of these experiments, tubulin was used to ensure equal sample loading.

These upon results indicate that application of NO donors and the overexpression of *AtNOA1* or *AtNIA2* stimulated HSPs expression in a *cngc6* mutant, providing further evidence that NO acts downstream of CNGC6 in the HS pathway.

### Effects of NO on the activity of Ca^2+^-permeable channel

The above results provided evidence of the role of CNGC6 on the NO-mediated acquisition of thermotolerance in Arabidopsis seedlings. In plants, a specific role for NO in controlling Ca^2+^ homeostasis has also been reported [24].

**Fig. 7:**
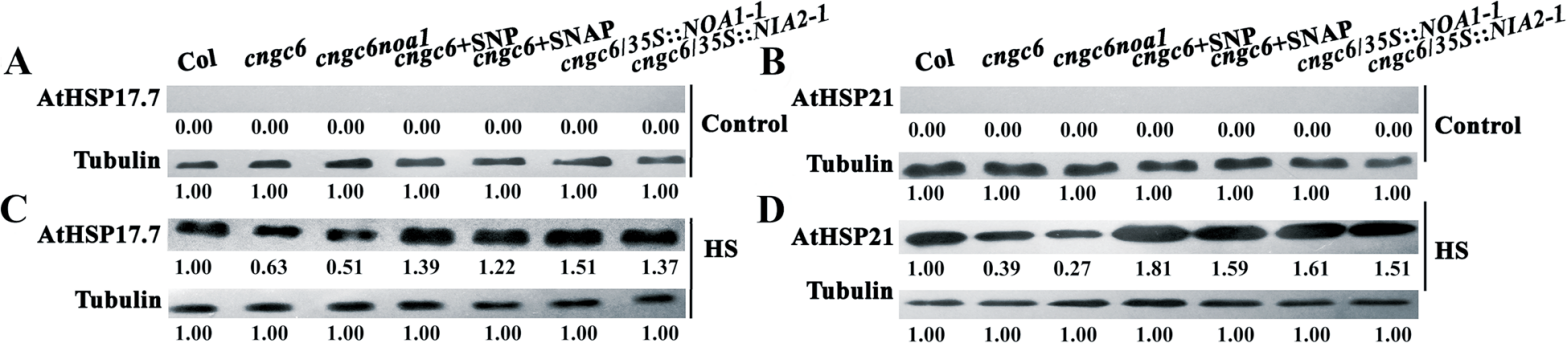
Patch-clamp analysis of Ca^2+^-permeable channels in wild-type, cngc6, noa1, cngc6/35S::NOA1–1, and cngc6/35S::NIA2–1 seedlings. The Ca^2+^ current before HS (at 22 °C, control) and after HS (at 37 °C, HS) was compared in the root cell protoplasts of 10-day-old wild-type (**a**), *cngc6* (**b**), *noa1* (**c**), *cngc6*/*35S*::*NOA1*–*1* (**d**) and *cngc6*/*35S*::*NIA2*–*1* (**e**) plants. The Ca^2+^ current was recorded by step voltage clamp. Each trace is a representative current from six protoplasts. Currents in the protoplasts are shown in the left and middle columns, respectively. The *I–V* curve is shown in the right column (mean ± SD, n = 6)

To determine whether NO affects the function of heat-responsive Ca^2+^-permeable channels under HS conditions, we examined the effects of endogenous NO on the activity of CNCG6 in the PM of Arabidopsis root protoplasts with the whole-cell patch-clamp technique [20]. Under normal conditions, the Ca^2+^ current in *cngc6* (− 36 pA) was lower than that in wild type (− 118 pA) at − 200 mV. Under HS at 37 °C, the inward Ca^2+^ current was greatly increased to − 369 pA in wild type within 1 min, whereas only a slight increase (to − 90 pA) was observed in *cngc6* (Fig. 7a, b), consistent with our previous report [20]. In *noa1* mutant with low internal NO levels, the Ca^2+^ currents showed no obvious difference with those in wild type under normal and HS conditions (Fig. 7c). In two transgenic lines with high internal NO levels, *cngc6*/*35S*::*NOA1*–*1* and *cngc6*/*35S*::*NIA2*–*1*, the Ca^2+^ currents were similar to those of *cngc6* (non-transgenic background) under normal and HS conditions (Fig. 7d, e). These results were proved by the determination of [Ca^2+^]_cyt_ level using a Ca^2+^ sensor protein aequorin (Additional file 1: Figure S1), suggesting that NO had no obvious effect on Ca^2+^ channel activity.

## Discussion

### The relationships among CNGC6, Ca^2+^, and NO accumulation under HS conditions in Arabidopsis seedlings

Exposure to high temperatures usually results in increased [Ca^2+^]_cyt_ and the production of NO in plant cells; both of Ca^2+^ and NO play important roles in plant resistance to heat stress [10, 26, 29]. What is the relationship between Ca^2+^ and NO signaling pathways in heat tolerance? In this study, we obtained evidence that CNGC6, a heat-activated Ca^2+^-permeable channel, induces NO production so as to regulate the accumulation of HSPs to promote thermotolerance in Arabidopsis.

NO, as an important messenger in multiple biological processes in plants, is induced by numerous components to mediate resistance responses. We previously found that NO functions as a signal in plant thermotolerance [10]. We subsequently demonstrated that H_2_O_2_ functions as a second messenger in the induction of thermotolerance through NO [12]. H_2_O_2_ was previously reported to be involved in increasing intracellular levels of free Ca^2+^ [30]. Recently, several studies have highlighted the role of Ca^2+^ in initiating NO production in plants [25, 31]. Thus, we deduced that there should be a close relationship between Ca^2+^ and NO in HS signaling.

CNGCs affect various biological processes by regulating the influx and efflux of ions. We identified a heat- and cAMP-activated PM Ca^2+^ channel, CNGC6, which is involved in conducting Ca^2+^ into the cytoplasm; in that study, a T-DNA insertion mutant, *cncg6*, exhibited a lower Ca^2+^ current than wild type [20]. Moreover, the inward Ca^2+^ currents in COM12 and OE8 were higher than that in *cngc6* depending on the *CNGC6* expression level under HS conditions [20], suggesting that CNGC6 mediates the influx of Ca^2+^ into cells. Therefore, we utilized *cngc6* and the above two transgenic lines to investigate the relationship between CNGC6 and NO under HS conditions.

First, we examined NO accumulation using the fluorescent probe DAF-FM DA under HS conditions. Under normal growth conditions, the NO levels of wild-type, *cngc6*, COM12, and OE8 seedlings were relatively stable. However, high temperature exposure stimulated NO accumulation in these seedlings according to their *CNGC6* expression levels (Fig. 1a, b), suggesting a significant role for CNGC6 in thermotolerance via the regulation of NO accumulation.

Due to the biological activity of CNCG6 in HS-treated plants, we examined the effect of Ca^2+^ on NO accumulation in wild-type, *cngc6* and OE8 plants. Our experiments show that Ca^2+^ stimulated the accumulation of NO in these seedlings under HS conditions. However, the Ca^2+^ chelator EGTA obviously inhibited NO accumulation in wild-type and OE8 seedlings (Fig. 1c, d), suggesting that CNGC6-mediated free Ca^2+^ is a key element in promoting NO signaling. Thus, we propose that CNGC6 regulates internal NO levels via free Ca^2+^ under HS conditions.

### Effects of NO and CNGC6 on thermotolerance in Arabidopsis seedlings

To clarify the effects of NO and CNGC6 on heat tolerance, we examined the effects of NO on the survival of *cngc6* plants under HS conditions. A moderate concentration (20 μM) of two NO donors, SNP and SNAP, elevated the internal NO level and the survival ratio of HS-treated *cngc6* seedlings (Fig. 2). The overexpression of two NO synthesis-related enzymes, *NOA1* and *NIA2*, enhanced the internal NO level and the survival ratio of their transgenic lines, respectively, compared to *cngc6* (non-transgenic background) under HS conditions (Figs. 3, 4). These results indicate that a rise in internal NO rescued the heat susceptibility of the plants due to the removal of *CNGC6*.

Finally, we obtained the double mutant *cngc6 noa1*, which exhibited a phenotype similar to that of *noa1* under normal growth or HS conditions (Fig. 5), indicating that deficiencies in both *noa1* and *CNGC6* do not potentiate the heat susceptibility caused by a deficiency in *noa1* alone.

The HS response is mediated via a process in which HSP expression is induced [1]. To confirm the relationship between CNGC6 and NO in the HS signaling pathway, we examined the effects of CNGC6 and NO on HSP expression under HS conditions. HSP genes are classified based on their molecular masses, such as HSP100, HSP90, HSP70, HSP60, and small HSPs. Among them, the small HSPs are the most important ones due to their crucial roles in plant survival under HS conditions [32]. Herein, two small HSPs, HSP17.7 and HSP21, were used to confirm whether CNGC6 through NO promotes HSP expression in plant thermotolerance. Western blotting revealed that under HS conditions, the reduced *CNGC6* level in *cngc6* inhibited the expression of AtHSP17.7 and AtHSP21. On the contrary, NO donors and *NOA1* and *NIA2* overexpression in *cngc6* mutant increased the expression of AtHSP17.7 and AtHSP21 (Fig. 6), indicating that CNGC6 stimulates HSP expression through NO.

Collectively, our data provide physiological, pharmacological and biochemical proof of the existence of a HS signaling pathway in which NO acts as a downstream partner of CNGC6 to confer thermotolerance.

### Effects of NO on Ca^2+^ fluxes in thermotolerance in Arabidopsis seedlings

NO-induced cGMP stimulates the synthesis of the NAD^+^ metabolite cADPR, which is a potent Ca^2+^-mobilizing agent that binds to intracellular Ca^2+^ channels and activates the release of Ca^2+^ in tobacco (*Nicotiana tabacum*) [33]. In addition, a specific role for NO in elevating the availability of intracellular and extracellular Ca^2+^ pools was assumed during auxin-induced adventitious root formation in cucumber (*Cucumis sativus*) [34]. Accordingly, we wondered whether NO alters Ca^2+^ fluxes to affect thermotolerance.

A significant increase in [Ca^2+^]_cyt_ was observed in response to a single temperature increase from 22 to 37 °C in wild-type plants (Fig. 7). However, the current was obviously inhibited in *cngc6*, *cngc6*/*35S*::*NOA1*–*1*, and *cngc6*/*35S*::*NIA2*–*1* plants but not clearly altered in *noa1* plants (Fig. 7), indicating no clear effect of NO on Ca^2+^-permeable channel activity. These data, in combination with the results shown in Figs. 2, 3, 4 and 5, suggest that the HS-induced change in [Ca^2+^]_cyt_ unidirectionally influences NO signaling in plants. A plausible explanation for these results is that supplementation with NO, a downstream molecule, restored the heat-sensitive status of the *CNGC6*-deficient seedlings (Figs. 2, 3, 4 and 5) but could not activate the heat-responsive activity of CNGC6 (Fig. 7).

However, there was a strange phenomenon that in the *cngc6* mutant, NO production was also stimulated under HS, which can be slightly inhibited by EGTA (Fig. 1). This should be due to the action of phosphoinositide-specific phospholipase C9 [35] and phosphoinositide-specific phospholipase C3 [36] in mediating the elevation of [Ca^2+^]_cyt_ through intracellular calcium pool under HS.

## Conclusion

**Fig. 8:**
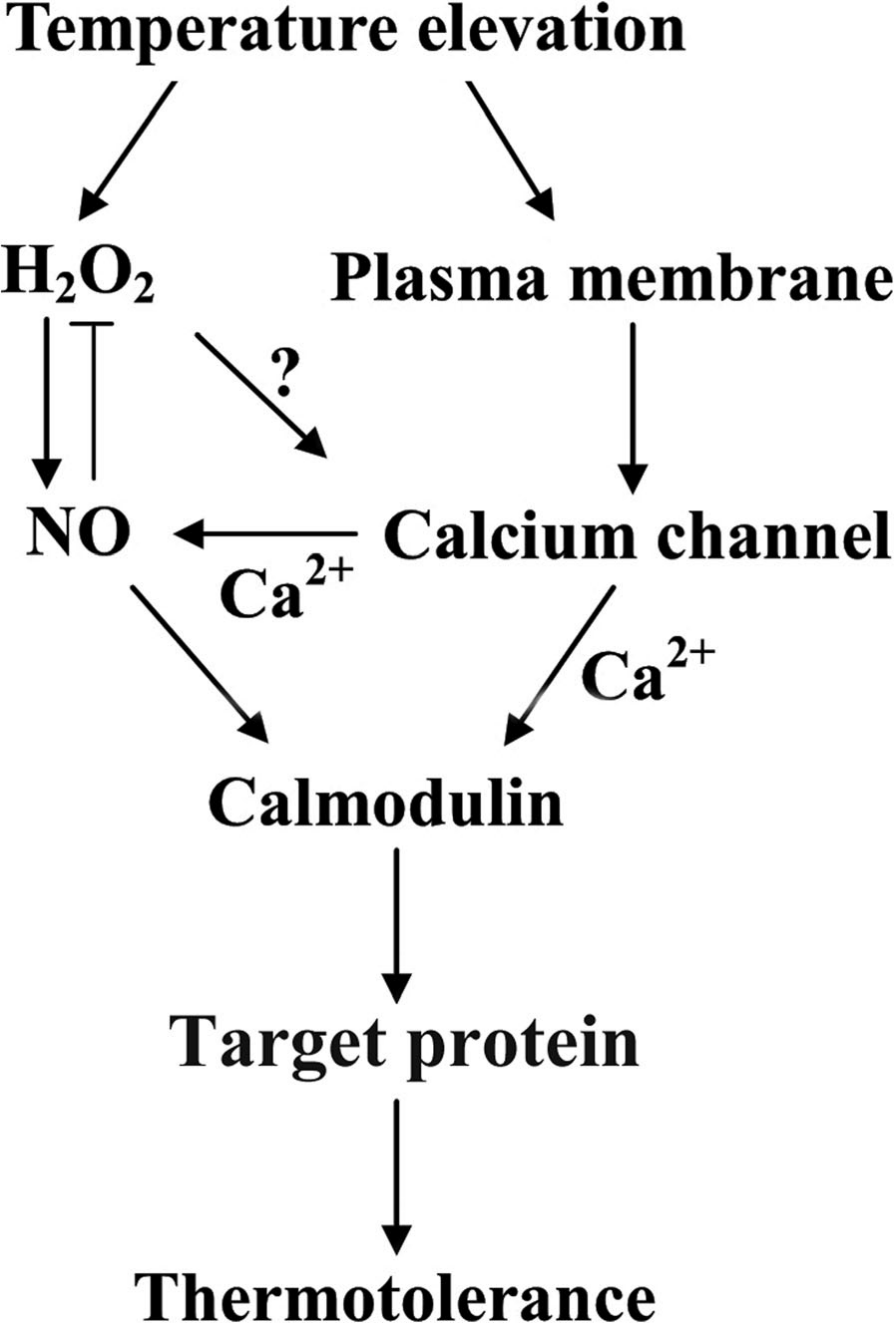
Model of the involvement of CNGC6 in the NO pathway of HS signal transduction. Black arrows indicate pathways supported by evidence; the t-shaped straight line indicates a repressive effect. The question mark indicates an unknown player

To our knowledge, these data provide the evidence that the HS-responsive Ca^2+^-permeable channel CNGC6 participates in the induction of HS signaling through NO. We previously proposed a model for HS signaling in which the HS signal was identified by an unknown receptor, leading to an increased H_2_O_2_ level, which directly stimulated NO accumulation and activated AtCaM3 to initiate plant adaptation to high temperatures [12]. Additionally, a feedback inhibition was proposed to exist between NO and H_2_O_2_ in the HS pathway in Arabidopsis seedlings [14]. In this study, CNGC6 was found to act upstream of NO through free Ca^2+^ in the response of plants to HS. H_2_O_2_ application was also shown to increase the intracellular level of free Ca^2+^ [37]. Ca^2+^ and AtCaM3 are involved in the expression of HSP genes in Arabidopsis [38]. CaM, upon binding to Ca^2+^, attaches to specific target proteins, altering their functions as part of a HS-responsive Ca^2+^ signal transduction pathway; demonstrated targets include CaM-binding protein kinase 3 [39] and PP7 [40]. These findings suggest that complicated relationship occurs among H_2_O_2_, NO, Ca^2+^ channels, and the Ca^2+^/CaM-dependent activation of target proteins in the HS pathway (Fig. 8).

## Methods

### Plant materials and growth conditions

The wild-type and mutant Arabidopsis were Col-0 ecotype. The *noa1* seeds were provided by Dr. Nigel M. Crawford (University of California, San Diego, CA). *35S*::*NIA2–1* and *35S*::*NIA2–3* seeds were obtained from Dr. Chun-Peng Song (Henan University, Kaifeng, China). The double mutant *cngc6 noa1* and transgenic lines *cngc6/35S*::*NIA2–1* and *cngc6*/*35S*::*NIA2–3* were obtained by crossing, while the transgenic lines *cngc6*/*35S*::*NOA1–1* and *cngc6*/*35S*::*NOA1–2* were obtained by the floral dip method.

The Arabidopsis seeds were surface-sterilized in 2% (v/v) sodium hypochlorite for 1 min and then washed thoroughly with water. The sterilized seeds were placed on Murashige and Skoog (MS) medium containing 3% sucrose and 0.7% agar and kept at 4 °C in the dark for 48 h. The plants were then transferred to a growth chamber set at 22 °C and 120 μmol m^− 2^ s^− 1^ on a 16-h 16-h daily light period.

For chemical treatment, 2 ml of SNP or SNAP (20 μM each) (Sigma-Aldrich, St. Louis, MO) were sprinkled onto the leaf surfaces of 8-day-old seedlings after filter sterilization for 48 h. Water was used as a substitute for control seedlings. After 8 h of pretreatment, the seedlings were subjected to HS conditions [12]. In addition, 5 mM CaCl_2_ or 2 mM EGTA was used to pretreat wild-type, *cngc6*, and OE8 seeds for 30 min in the fluorescence experiment, with ultrapure water as the control.

### Thermotolerance testing

Eight-day-old seedlings, grown at 22 °C, were incubated in sterilized 5 mM CaCl_2_ at 37 °C for 30 min, returned to 22 °C for 2 h, then challenged at 45 °C for 100 min, and finally returned 22 °C for 5 days of recovery [26]. Those seedlings being still green and continuing to produce new leaves were registered as survivors. For Western blotting, 10-day-old seedlings were kept at 37 °C for 2 h and collected for the analyses of HSP accumulation.

All the experiments were repeated for at least three times, and three independent biological replicates for each time.

### Fluorescence microscopy

NO was visualized using the specific fluorescent probe DAF-FM DA (Sigma-Aldrich) as described previously [5] with some modifications. Wild-type and mutant seedlings were incubated in 1 ml of liquid MS medium (pH 5.8) with 10 μM DAF-FM DA for 20 min. Then, the roots were washed three times for 15 min each in liquid MS medium prior to visualization with a fluorescence microscope (Eclipse TE 200, Nikon, Tokyo, Japan). The signal intensities were calculated using MetaMorph (Molecular Devices, Sunnyvale, CA).

### Vector construction and the generation of transgenic plants

To obtain *AtNOA1-YFP* for the production of plants overexpressing *AtNOA1* in a *cngc6* background, *AtNOA1* complementary DNA (cDNA) was amplified by reverse transcription-polymerase chain reaction (RT-PCR) with the primers NOA1F1 (5′-**CACC**ATGGCGCTACGAACACTCTCAA-3′) and NOA1F2 (5′-AAAGTACCATTTGGGTCTTACT-3′) (the underlined sequence in NOA1F1 was used to link to pENTR/SD/D-TOPO within this reaction chain). The product was cloned in the sense orientation into pENTR/SD/D-TOPO and then into pEarleyGate 101 using Gateway LR Recombinase (Invitrogen Corp., Carlsbad, CA) to generate *35S*::*AtNOA1*-*YFP*.

The transformation of this construct into Arabidopsis (*cngc6*) was performed according to the floral dip method [41] with *Agrobacterium tumefaciens* (strain GV3101). Transformants were screened on plates containing 15 mg l^− 1^ of Basta. Homozygous T3 transgenic lines were selected for further analysis.

### RT-qPCR analysis

Total RNA (500 ng) was isolated from 10-day-old seedlings at 37 °C for 1 h with a PrimeScript RT Reagent Kit (Takara Bio Inc., Otsu, Japan) for first-stand cDNA synthesis as the manufacturer’s instructions. The program was as follows: initial polymerase activation for 10 s at 95 °C followed by 40 cycles of 95 °C for 5 s and 60 °C for 31 s. The reactions were performed using an ABI Prism 7000 sequence detection system (Applied Biosystems, Foster City, CA) with SYBR Premix Ex Taq (Takara Bio Inc.). Primer pairs were designed using Primer Express (Applied Biosystems). Detailed primer sequences are shown in Additional file 1: Table S1.

### Western blot analysis

Ten-day-old seedlings were kept at 37 °C for 2 h and then ground in liquid nitrogen. Total protein was extracted with an extraction buffer (10 mM HEPES, pH 7.9, containing 0.4 M NaCl, 0.5 mM dithiothreitol, 0.1 mM EDTA, 5% glycerol, and 0.5 mM phenylmethanesulfonyl fluoride), and the extracts were purified by centrifugation at 14,000 x *g* for 20 min at 4 °C. The supernatants were transferred to fresh tubes, and the protein content was measured as the description of Bradford [42]. Total proteins (50 μg) were analyzed by western blotting as described previously [12].

### Preparation of protoplasts and electrophysiology analysis

Protoplasts were isolated as described previously [43] from 1 cm long of root tips of Arabidopsis seedlings cultivated vertically at 22 °C for 6–7 days. Whole-cell voltage patch-clamping was carried out as described previously [20, 44].

### In vivo reconstitution of aequorin and Ca^2+^ measurement

In vivo reconstitution of the aequorin was conducted according to Gao’s method [20]. The [Ca^2+^]_cyt_ level was obtained by calculating the pCa with the equation as described previously [45].

## Additional files

Additional file 1: **Figure S1.** [Ca^2+^]_cyt_ analysis using Ca^2+^ sensor protein aequorin in wild-type, *cngc6*, *noa1*, *cngc6*/*35S*::*NOA1*–*1*, and *cngc6*/*35S*::*NIA2*–*1* seedlings. **Table S1.** Primers used for real-time quantitative RT-PCR. (PDF 91 kb)

## Data Availability

The datasets used and/or analysed during the current study are available from the corresponding author on reasonable request. Sequence data from this article can be found in the Arabidopsis Genome Initiative data library under the following accession numbers: *NOA1* (At3g47450), *NIA2* (At1g37130), *CNGC6* (At2g23980), and *Actin2* (At3g18780).

## Abbreviations

[Ca^2+^]_cyt_: Cytosolic Ca^2+^
Ca^2+^: Calcium ions
CaM3: Calmodulin3
cDNA: Complementary DNA
CNGC: Cyclic nucleotide-gated cation channels
DAF-FM DA: 4-amino-5-methylamino-2′,7′-difluorofluorescein diacetate
DAF-FM: 4-amino-5-methylamino-2′,7′-difluorofluorescein
H_2_O_2_: Hydrogen peroxide
HS: Heat shock
HSP: Heat shock protein
MS: Murashige and skoog
NIA1: Nitrate reductase 1
NIA2: Nitrate reductase 2
NO: Nitric oxide
NOA1: Nitric oxide-associated protein 1
NOS: NO synthase
NR: Nitrate reductase
PM: Plasma membrane
RT-PCR: Reverse transcription-polymerase chain reaction
RT-qPCR: Real-time quantitative RT-PCR
SDS-PAGE: Sodium dodecyl sulfate polyacrylamide gel electrophoresis
SNAP: *S*-nitroso-*N*-acetylpenicillamine
SNP: sodium nitroprusside
