# Supplementary material for: Cyclic nucleotide-gated ion channel 6 mediates thermotolerance in Arabidopsis seedlings by regulating nitric oxide production via cytosolic calcium ions
Source: BMC Plant Biol. 2019 Aug 20;19:368. doi: 10.1186/s12870-019-1974-9 (PMC6702746; doi:10.1186/s12870-019-1974-9)
Supplement: Supplementary file 1 — Figure S1. [Ca2+]cyt analysis using Ca2+ sensor protein aequorin in wild-type, cngc6, noa1, cngc6/35S::NOA1–1, and cngc6/35S::NIA2–1 seedlings. Table S1. Primers used for real-time quantitative RT-PCR. (PDF 91 kb) [file 12870_2019_1974_MOESM1_ESM.pdf]

Additional files

Figure S1. $[Ca^{2+}]_{cyt}$ analysis using Ca^{2+} sensor protein aequorin in wild-type, *cngc6*, *noa1*, *cngc6*/*35S*::*NOA1-1*, and *cngc6*/*35S*::*NIA2-1* seedlings.

Relative $[Ca^{2+}]_{cyt}$ in 10-day-old seedlings of transgenic Arabidopsis expressing aequorin incubated at 22°C (no HS) and 37°C (HS). The experiment was repeated three times with similar results.

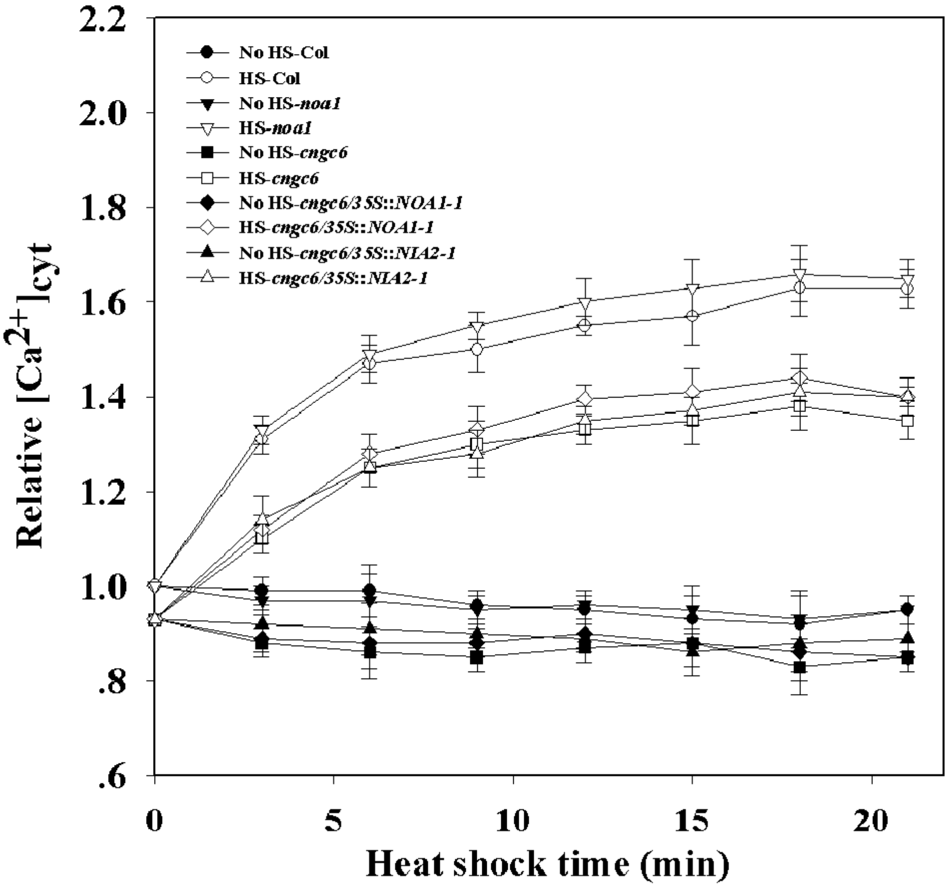

Table S1. Primers used for real-time quantitative RT-PCR

Primer name	Sequence (5'-3')
NOA1RT-F	ATAACATAACAACAAACAACAG
NOA1RT-R	GCTCTCACCCTTGGGACTAC
NIA2RT-F	GCAAGCCACACAAGGGAGAG
NIA2RT-R	CGCCATCCATCCACCAGAT
CNGC6RT-F	CCGCGGAGAGCTTGTTATAG
CNGC6RT-R	CCGGCAGTTCTCTTCAGTTC
Actin2RT-F	GGTAACATTGTGCTCAGTGGTGG
Actin2RT-R	AACGACCTTAATCTTCATGCTGC
